# Indocyanine green lymphography imaging of normal lymphatic drainage in the lower limbs

**DOI:** 10.1093/bjr/tqag008

**Published:** 2026-01-10

**Authors:** Mike Mills, Malou van Zanten, Greta Brezgyte, Bernard Ho, Julian Pearce, Stephanie Wilken-Smith, Manan Shelton, Peter Mortimer, Hiroo Suami, Kristiana Gordon, Pia Ostergaard

**Affiliations:** School of Health and Medical Sciences, City St George’s, University of London, London, SW17 0RE, United Kingdom; School of Health and Medical Sciences, City St George’s, University of London, London, SW17 0RE, United Kingdom; Department of Dermatology and Lymphovascular Medicine, Nij Smellinghe Hospital, Drachten, 9202 NN, The Netherlands; School of Health and Medical Sciences, City St George’s, University of London, London, SW17 0RE, United Kingdom; School of Health and Medical Sciences, City St George’s, University of London, London, SW17 0RE, United Kingdom; Lymphovascular Medicine, Dermatology Department, St George’s University Hospitals NHS Foundation Trust, London, SW17 0QT, United Kingdom; School of Health and Medical Sciences, City St George’s, University of London, London, SW17 0RE, United Kingdom; Lymphovascular Medicine, Dermatology Department, St George’s University Hospitals NHS Foundation Trust, London, SW17 0QT, United Kingdom; School of Health and Medical Sciences, City St George’s, University of London, London, SW17 0RE, United Kingdom; Lymphovascular Medicine, Dermatology Department, St George’s University Hospitals NHS Foundation Trust, London, SW17 0QT, United Kingdom; School of Health and Medical Sciences, City St George’s, University of London, London, SW17 0RE, United Kingdom; School of Health and Medical Sciences, City St George’s, University of London, London, SW17 0RE, United Kingdom; Lymphovascular Medicine, Dermatology Department, St George’s University Hospitals NHS Foundation Trust, London, SW17 0QT, United Kingdom; Australian Lymphoedema Education, Research & Treatment (ALERT) Centre, Faculty of Medicine, Health and Human Sciences, Macquarie University, Sydney, New South Wales, NSW 2109, Australia; School of Health and Medical Sciences, City St George’s, University of London, London, SW17 0RE, United Kingdom; Lymphovascular Medicine, Dermatology Department, St George’s University Hospitals NHS Foundation Trust, London, SW17 0QT, United Kingdom; School of Health and Medical Sciences, City St George’s, University of London, London, SW17 0RE, United Kingdom

**Keywords:** indocyanine green lymphography (ICGL), near-infrared fluorescence (NIRF), lymphatic system, healthy controls, lower limb

## Abstract

**Objectives:**

Indocyanine green lymphography (ICGL) has emerged as a potentially powerful tool for the study of the superficial lymphatic system and to support the diagnosis of lymphoedema. However, detailed descriptions of ICGL findings in healthy individuals are limited. In this study, we imaged a series of healthy participants using ICGL, attempting to establish quantitative and qualitative ICGL parameters of the lower limb.

**Methods:**

Sixteen healthy individuals aged 20-55 years were recruited to undergo lower limb ICGL after 0.1 mL injections of 1 g/L ICG were administered intradermally to 5 locations around the foot. Outcome measures included: (1) the drainage routes of contractile lymphatic collectors observed, (2) the number of lymphatic vessels crossing the anterior ankle, and (3) the pumping frequency of lymphatic vessels. Abnormal features, such as highly tortuous or vessels with retrograde lymph flow, were noted.

**Results:**

Propulsion of ICG containing lymph could be seen in all individuals, with drainage via the anteromedial and anterolateral drainage pathways predominating (observed in 31/32 and 25/32 limbs, respectively). The number of lymphatic vessels crossing the anterior ankle was 3.4 ± 1.1 with an average rate of 1 propulsion every 66 seconds in the vessels investigated. Isolated cases of highly tortuous and refluxing vessels were observed.

**Conclusions:**

Although limited by absorption and scatter of infrared light, ICGL facilitated the characterization of normal lower limb lymphatic vessels through a rigorous set of objective measures. This in turn will allow better identification of pathological changes.

**Advances in knowledge:**

Establishment of normal lower limb lymphatic anatomy and function.

## Introduction

The lymphatic system has historically been challenging to image, which has limited the understanding of the lymphatics and their contribution to disease. Comprised predominantly of fragile, sub-millimetre vessels, filled with colourless fluid, lymphatic cannulation, and delivery of contrast agents for radiographic lymphangiography is technically demanding with potentially severe side effects including pulmonary embolism.^1^ Lymphatic flow is also slower than blood circulation and erratically pulsatile, making techniques such as Doppler ultrasound not generally applicable to the study of the lymphatics.^2^

Indocyanine green (ICG) lymphography (ICGL) is an imaging technique which offers higher spatial and temporal resolution visualization of superficial lymphatics compared to the more routinely used lymphoscintigraphy.^3^ Relying on preferential trafficking of the ICG into lymphatic vessels following interstitial injection, ICGL does not require direct lymphatic cannulation.^2^ Illumination with near infrared (IR) radiation, and detection of the shifted IR emission, may also be performed with small portable devices providing immediate depiction of the lymphatics. ICGL can therefore be used in the clinic to allow real time and *in vivo* imaging of the superficial lymphatic vessels with no ionizing radiation exposure. Results including the identification of lymphatic pathways, and whether the flow within them is functional or disrupted, can then guide and assess therapies for lymphoedema such as manual lymphatic drainage and lymphovenous anastomosis surgery.^4^^,^^5^

ICGL provides valuable novel insights into the functioning of the superficial lymphatic system. Previous studies have used ICGL to study the lymphatics in the extremities of patients with primary and secondary lymphoedema and demonstrate benefits over alternative methods of investigation.^6–10^ To recognize pathological abnormalities, one must know what is normal, and yet few reports of lymphatic vessels within normal legs of healthy volunteers have been published. Many studies have imaged the non-oedematous limb of unilateral lymphoedema patients as a comparator to an affected limb, however these limbs could conceal incipient lymphoedema, while studies of cadavers cannot capture true lymphatic physiology.^11–13^

In this study, we aim to provide normal parameters for the anatomy and physiology of the superficial lymphatic vessels in the lower limbs of healthy individuals without clinical symptoms of lymphatic disease using ICGL.

## Methods

### Participants

Healthy volunteers recruited to undergo ICGL of the lower limbs at City St George’s, University of London, between December 21, 2022 and November 21, 2023 had their ICGL data reviewed in this observational study. Participants included staff members recruited following an internal recruitment email, and friends or partners of existing patients, but not their blood-related relatives. Prior to enrolment participants were screened for any history of lymphatic disease and, to ensure their safety to undergo ICGL, any known allergy to ICG, sodium iodide or iodine. Pregnant individuals were also not imaged.

Ethics approval for this research was granted by the Southeast Coast – Brighton & Sussex Research Ethics Committee, 14/LO/0753.

### ICG contrast injection protocol

**Figure 1.**
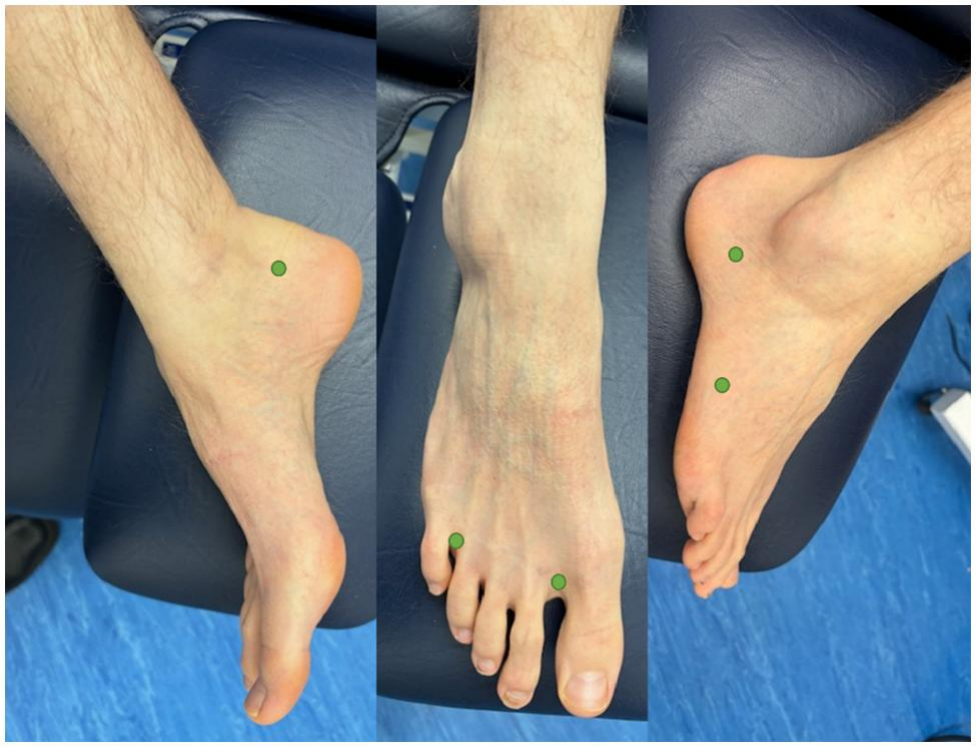
The 5 ICG injection points in the foot (green dots) shown on a non-oedematous foot. The 3 frames show the injection locations of the medial rear-foot (left frame), first and fourth web spaces of the forefoot (middle frame) and the lateral midfoot and rear-foot (right frame). Adapted with permission from Brezgyte et al^33^ and subsequently published under a CC-BY 4.0 licence in Brezgyte et al^10^.

To facilitate ICG uptake into each of the 4 lymphatic vessel groups of the lower limbs (anteromedial, anterolateral, posterolateral and posteromedial), ICG was administered into 5 circumferential locations in each foot: first and fourth web spaces, lateral midfoot and lateral and medial rear-foot (Figure 1).^14^^,^^15^ Each injection consisted of 0.1 mL of a 1 g/L mixture comprising 25 mg of ICG (Verdye) dissolved into 20 mL water for injection and 5 mL 1% lidocaine. ICG was administered manually through intra-dermal injections using a 29G needle and 1 mL syringe. Following injections, participants were asked to perform foot and toe flexions 10 times before immediate commencement of imaging.

### Imaging protocol

ICGL was performed with a Fluobeam 800 (Fluoptics, Grenoble, France), capable of exciting the area under investigation with IR radiation at 750 nm and detecting the emitted IR with a single optical head. The optical head was used either freehand or mounted on an articulating arm to improve camera stability whilst imaging. A distance of 15-20 cm between the optical head and the skin was generally maintained as per the manufacturer’s guidance. Moving beyond this range was acceptable if the operator believed they could achieve improved focus or sensitivity by doing so. The operator also had control of the infrared exposure time and background light level emitted by the optical head, however, an exposure time ≤40 ms was generally employed as this ensured the maximum achievable image frame rate of 25 frames per second. Focussing was automatically performed by the imaging system. No offline processing of recorded still or video image data was performed.

Imaging consisted of a mobile phase to document the overall pattern of lymphatic enhancement within a limb and separate videos of 3-5 minutes focusing on, for example, the anterior ankle as well as other anatomical regions where lymphatics could be identified. The optical head was mounted in the articulated arm during all video capture.

Checking the integrity of 1-way lymphatic collector valves by the application of manual pressure down the limb was routinely performed, as was imaging the rear of the limb with the participant standing if they could comfortably do so.

### Image analysis

Employing the classification described by Shinaoka et al^14^^,^^15^ lymphatic vessels were categorized based on the path taken in the limb as belonging to 1 of 4 groups: anteromedial, posteromedial, anterolateral, and posterolateral. Additional imaging measures of interest were: (1) the number of lymphatic vessels crossing the anterior ankle (approximately at the level of the talus), (2) the pumping frequency within one of these ankle vessels (ie, the number of times a vessel emptied during continuous video acquisitions of at least 3 minutes, divided by the video duration) and (3) imaging signs considered of an abnormal nature.

Potentially abnormal imaging features included lymphatic vessels that were highly tortuous or directed across, or back down, the limb, the presence of lymph nodes, regions of dermal backflow and lymph stasis (ie, not being drained from the injection site). Instances where lymph was manually drainable back down the limb were also considered abnormal.

Notes taken during the imaging examination formed the backbone of the analysis. These records, and the corresponding images/videos from the ICGL session, were then interrogated further by an imaging scientist with 6 years of experience in lymphatic imaging (MM) to ensure the accuracy of the details recorded in the notes and to supplement where details were lacking. At this review stage, estimates of lymphatic pumping frequency in a single anterior ankle lymphatic of each limb were also made. In addition, in 10 healthy control ICGL videos where the average pumping rate closely matched that of the entire dataset, lymphatic pumping frequency was measured again by a second reviewer with less than a year of ICGL experience (SWS). The video and vessel to study was shared between reviewers but measurements were performed independently.

## Results

Sixteen healthy individuals aged between 20 and 55 years (7 females, mean age = 38.1 years, median age = 37.5 years; 9 males, mean age = 39.4 years, median age = 42.9 years) were recruited and imaged (see Table S1). Lymphatic vessels actively transporting ICG towards the groin were seen in all imaged limbs, and no adverse effects were recorded during or after imaging.

### Lymphatic anatomy

**Figure 2.**
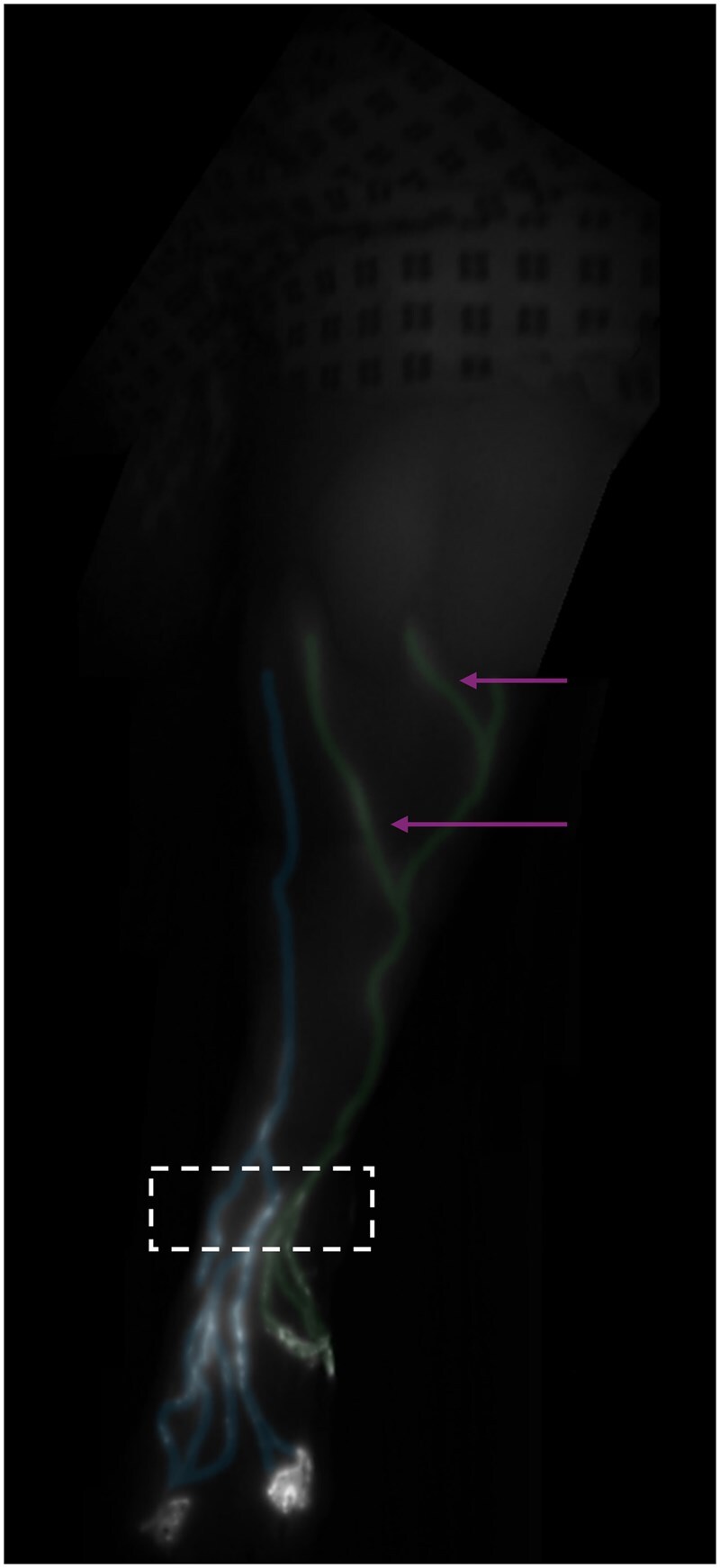
Representative indocyanine green lymphography image of a 41-year-old healthy female’s left lower limb. The location of injection sites can be seen at the bottom of the image, with the ICG travelling up towards the thigh at the top of the image. The anteromedial lymphatic vessels (highlighted with blue) originate from indocyanine green (ICG) injections in the first and fourth web spaces. Anterolateral lymphatic vessels (highlighted with green) can also be seen and originate from the ICG injection into the lateral midfoot. Four lymphatic vessels were observed passing the anterior aspect of the ankle (hashed box). Note that some lateral vessels move across to the medial limb just below the level of the knee (arrows).

**Figure 3.**
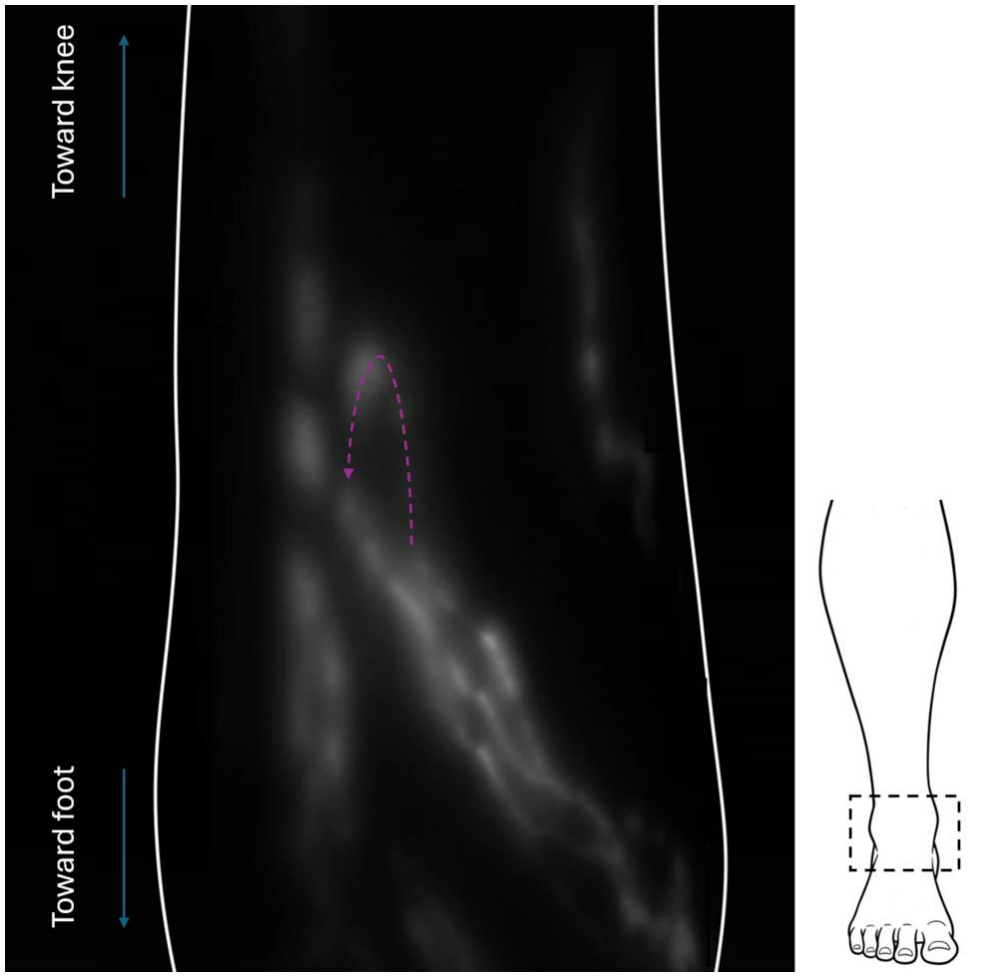
Indocyanine green lymphography image of the ankle of a 51-year-old healthy female, in which a vessel is seen to loop back on itself before merging with another vessel travelling up the leg. There is therefore a region of reverse flow, where fluid motion is down towards the foot (direction of flow indicated by the purple arrow) and can be observed in Video S1. The schematic on the right shows the approximate location of the imaged region (hashed box) and the limb outline is shown in white.

**Figure 4.**
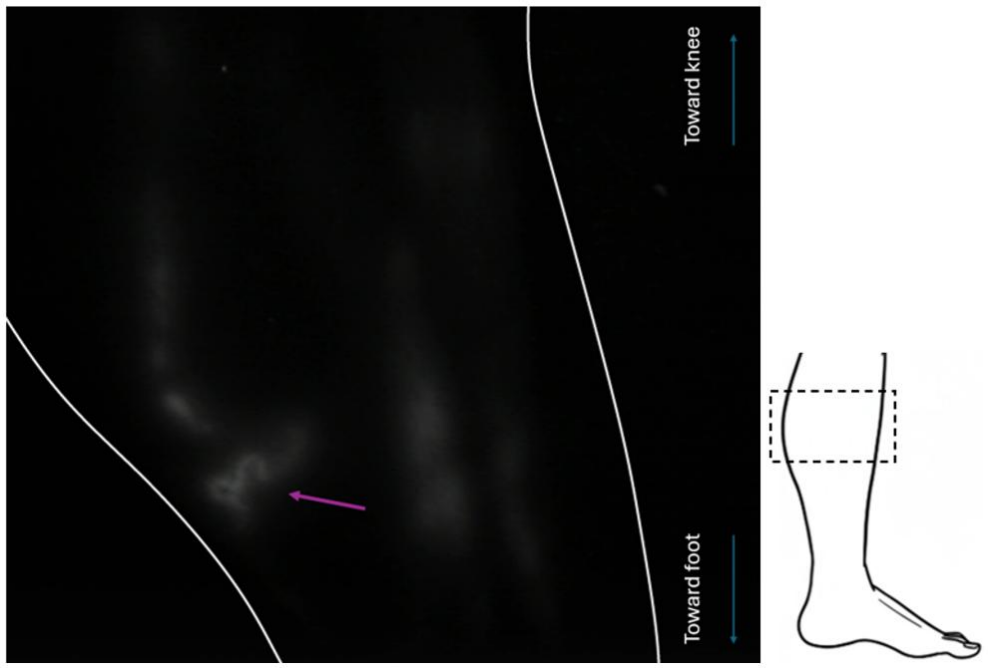
Small tortuous vessel segment located at the medial calf in a healthy individual (51-year-old female, the same individual as shown in Figure 3). Lymphatic vessels with a curvilinear nature were commonly observed as they travelled up the limb, but such tightly tortuous vessels (purple arrow) were identified only in this limb. As before, the schematic on the right shows the approximate location of the imaged region at the medial aspect of the leg (hashed box) and the limb outline is shown in white.

Contractile linear lymphatic vessels were observed in all 32 limbs of the enrolled participants (Figure 2). Vessels were traceable from injection site up to the groin in 87% of cases where thigh imaging was performed (27/31 limbs; in 1 limb thigh imaging was not performed). Regions of dermal backflow, lymph stasis and reflux were not observed. In one limb (3%), a vessel looped back on itself and pointed down the leg towards the foot before appearing to merge with another vessel travelling up the leg (Figure 3, Video S1). In 2 limbs (6%), discontinuous lymphatic vessels were demonstrated, comprising seemingly unconnected vessel fragments. In a separate limb (3%) an unusually tortuous lymphatic vessel fragment was evident (Figure 4).

Anteromedial and anterolateral pathways were observed in 97% (31/32) and 78% (25/32) of limbs, respectively (Figure 2), and all limbs had at least one, and at most 5, lymphatic vessels observed passing the anterior aspect of the ankle (mean ± standard deviation = 3.4 ± 1.1; median and mode = 4, interquartile range = 1).

**Figure 5.**
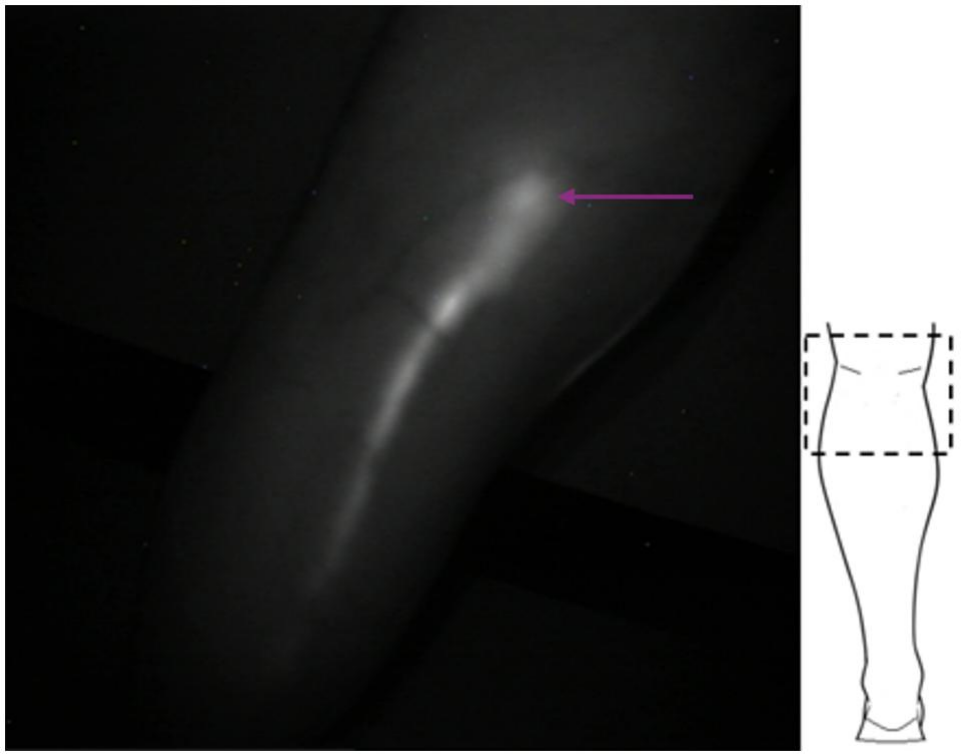
Posterolateral lymphatic vessel transporting indocyanine green from injection sites in the foot (unseen below frame) up the rear of the leg towards a lymph node (arrow) in the popliteal fossa of a 23-year-old male volunteer. No efferent vessels from the node into the thigh (upper portion of the image) could be observed. The schematic of the rear of the limb shows the approximate location of the imaged region (hashed box).

In all but 3 cases (29 of 32 limbs) the posterior aspect of the leg (calf) was imaged. Posteromedial enhancement was noted in 18 limbs. Posterolateral pathways were only noted in 9 limbs, 7 of which also showed posteromedial vessels. Popliteal vessels were detected in 13 limbs, and a popliteal node was detected in 12 (Figure 5).

Of the 20 limbs where it was performed, in both limbs of the same participant was ICG manually drainable back down the limb, suggestive of valve incompetence (Video S2). This was not observed in any of the remaining 18 limbs.

Details of individual vessel pathways and vascular features can be found in Table S1.

### Lymphatic pumping

An average pumping frequency of 0.9 ± 0.4 min^−1^ (range = 0.0-2.0 min^−1^) was estimated in anterior ankle lymphatics from videos lasting 180-250 seconds with 0-7 (mode and interquartile range = 2, median = 3) pumping events observed.

A subset of 10 videos was selected for repeat measurement by a second reviewer. Measurements obtained by the initial reviewer for these 10 resulted in an average frequency of 0.9 ± 0.5 min^−1^ (range = 0.0-1.4 min^−1^), well matching the overall group mean. The second reviewer meanwhile recorded a pumping frequency of 1.0 ± 0.6 min^−1^ (range = 0.0-1.4 min^−1^). An identical number of pumping events, and hence pumping frequency, was measured in 7/10 of the ICGL videos across reviewers. In the remaining 3 videos, the second reviewer recorded −1, +1, and +2 pumping events when compared to the initial measurements.

## Discussion

ICGL has emerged as an important technique for studying the superficial lymphatic system. It is particularly useful for studying vessels too small to observe with magnetic resonance imaging (MRI) or lymphoscintigraphy (LS) and has been deployed in previously difficult regions to image such as the genitals.^16–18^ Its advantages are real time and *in vivo* visualization of lymphatic transport with imaging devices that are relatively inexpensive and mobile. ICGL’s clinical applicability is enhanced further by its minimally invasive and non-ionizing nature. However, like any tracer-based technique, it is limited by possible contraindications to injection of a foreign agent and pain at administration. The poor penetrance of the infrared light also makes imaging vessels below around 2 cm in the skin impossible and the acquisition of image data is highly user dependent. Despite the limitations, ICGL has a role to play in the early detection and monitoring of lymphatic abnormalities, treatment planning, and assessment.^9^^,^^19^^,^^20^

Our recent review highlighted inconsistencies in reporting imaging details in ICGL studies and a lack of information regarding imaged participants however.^10^ Study protocols were also heterogenous, for example, some authors report delaying imaging by ≥1 hour after ICG injection whilst many image immediately such as we have here. ICG may also only be injected to the forefoot,neglecting injections around the foot.^10^ Standardization of injection and image acquisition protocols and common reporting guidelines, such as those developed by Suami et al^9^ or Shinaoka,^21^ may facilitate better comparison between studies and improve the diagnostic accuracy and clinical utility of ICGL.^9^^,^^22^

In this observational study, we focused exclusively on the imaging of healthy subjects without a history of lymphatic dysfunction, attempting to establish normal lymphatic anatomy and function. Imaging healthy controls, and not the contralateral limb of unilaterally affected lymphoedema patients where subclinical disease may be present,^12^^,^^23^ is essential for establishing normative ICGL data, which will facilitate improved sensitivity in the detection of lymphoedema, particularly in its early stages.

We acknowledge that our sample size was limited (*n* = 16) but believe the broad age range and sex mix of the included participants has led to the generation of meaningful results within a control group and to which patient data could be compared.

### Normal lymphatic drainage

The sites for administration of ICG were chosen to induce drainage via each of the 4 pathways described by Shinaoka et al.^14^^,^^15^ While vessels from the anterior injection sites, the anteromedial and anterolateral, were seen in all individuals, vessels following the posteromedial and posterolateral pathways were identified less frequently (18 and 9 limbs, respectively). Lymphatic vessels could be seen emanating from the posterior injection sites but often either followed a route more typical of the anteromedial and anterolateral lymphatics or were rapidly lost beyond the ankle. We suspect that the lower incidence of posterior lymphatic visualization results either from these vessels residing deeper within the limb than their anterior counterparts or preferential drainage via anterior routes in healthy individuals.

Despite the limited penetrance of the technique, lymphatic vessels could generally be detected all the way to the groin in our cohort. Within the thigh and groin, substantial scattering of the emitted light was evident, and the vessels faint and blurred, likely a result of the deeper course of the lymphatic vessels in the thigh compared to the lower leg. Functional assessment of the lymphatics in the lower limb is therefore likely to be most sensitive in the leg (below the knee) as opposed to the thigh, and justifies our choice to assess valve competency, vessel count and pumping frequency in the ankle and leg regions.

### Incidental findings and interpretation

Although infrequent, some features potentially indicative of lymphatic abnormality were observed in our healthy cohort. These included segments of tortuous lymphatic vessels, lymphatic flow oriented down a limb, and suspected valve incompetence in which ICG containing lymph could be manually pushed back down the limb. In order to be included in this study, participants had to report no history of lymphatic disease, however we cannot rule out the presence of sub-clinical lymphatic abnormalities or previous undisclosed injury being the cause of these findings. We believe, however, natural variations in individual lymphatic systems^9^^,^^24^^,^^25^ or limitations in the imaging technique (eg, insufficient IR penetrance leading to a discontinuous lymphatic appearance when vessels travel from superficial to deeper and back towards the skin again) to be the most likely explanation for these infrequent findings.

Popliteal nodes were observed in 40% of our healthy cohort when the popliteal fossa was imaged. The presence of popliteal lymph nodes in lymphoscintigrams has been reported as suggesting pathological rerouting of the radionuclide via the deep lymphatics.^26^^,^^27^ Lymphoscintigraphy is typically preceded by injections only to the forefoot which would not be expected to access the lymphatics at the rear of the limb in a healthy individual, unlike the ICG injections at the rear of the foot performed in this study.^14^ Clinicians and researchers therefore need to be cognizant of the injection sites for any dyes or tracers used in imaging studies and whether popliteal enhancement should be considered abnormal.

### Quantitative lymphatic assessment

An ability to not only detect but also quantify lymphatic dysfunction is obviously desirable and has been performed with lymphoscintigraphy for some time by measuring the proportion of radioactive tracer reaching efferent lymph nodes following a delay post tracer administration.^24^^,^^25^^,^^28^ Lymphoscintigraphy lacks the temporal and spatial resolution to monitor lymphatic propulsion in real time, however. Measurement of lymphatic contractile frequency and the speed of ICG bolus movement in the lymphatics have previously been attempted in the arms of healthy controls,^29^^,^^30^ as has the arrival time of ICG at the groin of lower limb lymphoedema patients following ICG injections in the foot.^31^ This shows the potential use of ICGL as a tool for quantifying lymphatic transport but, to our knowledge, no report has established similar measurements for the lower limbs in healthy individuals. Establishing normal ranges for quantitative parameters such as these would be beneficial for improving the detection of lymphatic abnormalities, particularly those subtle changes which are not yet manifesting clinically.^3^

Investigation of the anterior ankle enabled observation of linear vessels in all individuals, with 1-5 vessels visualized across all limbs. An average pumping rate of 0.9 min^−1^ was measured in the single vessel interrogated in each limb. This result is similar to that recorded in healthy forearms in the study of Lopera et al^30^ where an average of 0.9 min^−1^ pumping events also resulted, and at the dorsum of the hands in a study of lymphoedema patients by Granoff et al^32^ where 1.1 min^−1^ contractions were measured in the investigated vessels. Estimating pumping rate by eye was shown to be repeatable in this study, however, processing pipelines have been proposed to improve the efficiency and robustness of monitoring lymphatic contractions with ICGL.^29^^,^^30^^,^^32^ Even within these reports where some level of automation has been employed, only single vessels were investigated, however. The development of more advanced bolus tracking and image post-processing pipelines, perhaps leveraging artificial intelligence, may facilitate the measurement of multiple vessels within a video frame in an acceptable time, and reduce the effects of lessened sensitivity and apparent resolution for those lymphatic vessels residing at the periphery of the image frame.^29^^,^^32^

## Conclusion

Only by establishing normal parameters for anatomy, vessel numbers and characteristics of flow, can real pathological abnormalities be determined. ICGL is easy-to-use in the clinic and could be a valuable diagnostic tool if objective outcome measures, as developed in this study, are available. This would not only aid diagnosis of lymphoedema but help monitor disease progression. ICGL could also prove useful for the diagnosis of lymphatic abnormalities in previously understudied regions, such as the face, breast, and genital regions, and improve patient management by demonstrating measured responses to therapeutic interventions. However, factors such as optical scatter, participant motion and restricted light penetrance, reduce the quality of ICGL data and hence its interpretation.

Despite its limitations, healthy superficial lymphatic anatomy and functioning could be assessed *in vivo* and in real time in this study. Contractile superficial lymphatic vessels in each lower limb were observed in individuals with no history of lymphatic disease. We demonstrated that not all 4 lymphatic vessel groups of the lower limbs (anteromedial, anterolateral, posterolateral and posteromedial) will be observed in all cases, even with ICG injection sites chosen to access them. The lymphatic vessels which were observed were typically linear, with an average of 3 seen crossing the anterior ankle. Valve incompetency could be tested via the application of pressure down the limb and, along with tortuous lymphatic vessels, are generally absent in healthy legs. Tortuous vessels and valve reflux should therefore be considered abnormal, while depiction of popliteal lymphatic vessels and nodes should not when ICG is administered to the posterior of the foot.

## Supplementary Material

tqag008_Supplementary_Data
