# Supplementary material for: Indocyanine green lymphography imaging of normal lymphatic drainage in the lower limbs
Source: Br J Radiol. 2026 Jan 10;99(1179):577–82. doi: 10.1093/bjr/tqag008 (PMC13017491; doi:10.1093/bjr/tqag008)
Supplement: tqag008_Supplementary_Data [file tqag008_supplementary_data.zip › Supplementary_Table_1.docx]

Supplementary Table 1. Demographic and lymphatic vessel characteristics of the 32 limbs of the 16 healthy controls imaged with Indocyanine Green Lymphography. Note that the popliteal fossa was interrogated regardless of the presence of posterior lymphatic vessels seen originating from the foot as it was recognised that superficial structure in the popliteal fossa may be visible despite deeper lying posterior lymphatic not.
AM = Anteromedial; AL = Anterolateral; PM = Posteromedial; PL = Posterolateral; N = No, Y = Yes

| Limb # | Laterality | Sex | Age at Imaging (Years) | Pathways Observed | Popliteal Node? | Popliteal Vessel? | Tortuous Lymphatics? | Discontinuous Lymphatics? | Manually Drainable Down Limb |
| --- | --- | --- | --- | --- | --- | --- | --- | --- | --- |
| 1 | Left | Male | 44 | AM, AL | N | N | N | N | N |
| 2 | Right | Male | 44 | AM, AL, PM | N | Y | N | N | N |
| 3 | Left | Female | 38 | AM, AL, PM | N | N | N | N | N |
| 4 | Right | Female | 38 | AM, AL | N | Y | N | N | N |
| 5 | Left | Male | 43 | AM, AL, PM, PL | N | N | N | N | N |
| 6 | Right | Male | 43 | AM, AL | N | Y | N | N | N |
| 7 | Left | Male | 34 | AM, AL, PM | Not imaged | Not imaged | N | N | Not performed |
| 8 | Right | Male | 34 | AM, AL, PM, PL | Not imaged | Not imaged | N | N | N |
| 9 | Left | Male | 24 | AM, AL, PM | Y | Y | N | N | Not performed |
| 10 | Right | Male | 24 | AM, PM, PL | N | N | N | N | Not performed |
| 11 | Left | Male | 53 | AM, AL, PL | Y | N | N | N | Not performed |
| 12 | Right | Male | 53 | AM, AL, PM, PL | N | Y | N | N | N |
| 13 | Left | Female | 37 | AM, AL | Y | N | N | Y | Not performed |
| 14 | Right | Female | 37 | AM, AL | Y | Y | N | N | Not performed |
| 15 | Left | Female | 41 | AM, AL, PM | N | N | N | N | Not performed |
| 16 | Right | Female | 41 | AM, AL | N | N | N | Y | Not performed |
| 17 | Left | Male | 21 | AM, AL | N | N | N | N | Not performed |
| 18 | Right | Male | 21 | AM, PM | N | N | N | N | N |
| 19 | Left | Male | 47 | AM | N | N | N | N | Not performed |
| 20 | Right | Male | 47 | AM, AL | Y | N | N | N | N |
| 21 | Left | Female | 32 | AM, AL, PM | N | N | N | N | Not performed |
| 22 | Right | Female | 32 | AM, AL, PM, PL | Y | Y | N | N | Not performed |
| 23 | Left | Male | 56 | AM | Not imaged | Not imaged | Y | N | N |
| 24 | Right | Male | 56 | AM, AL, PM | N | N | N | N | N |
| 25 | Left | Male | 33 | AM, PM | Y | Y | N | N | N |
| 26 | Right | Male | 33 | AM, AL, PM, PL | Y | Y | N | N | N |
| 27 | Left | Female | 42 | AM | Y | Y | N | N | Y |
| 28 | Right | Female | 42 | AM | N | N | N | N | Y |
| 29 | Left | Female | 51 | AL, PM | N | N | N | N | N |
| 30 | Right | Female | 51 | AM, AL, PM | Y | Y | N | N | N |
| 31 | Left | Female | 26 | AM, AL, PL | Y | Y | N | N | N |
| 32 | Right | Female | 26 | AM, AL, PM, PL | Y | Y | N | N | N |
