# Supplementary material for: Indocyanine green lymphography imaging of normal lymphatic drainage in the lower limbs
Source: Br J Radiol. 2026 Jan 10;99(1179):577–82. doi: 10.1093/bjr/tqag008 (PMC13017491; doi:10.1093/bjr/tqag008)
Supplement: tqag008_Supplementary_Data [file tqag008_supplementary_data.zip › Supplementary_video_captions.docx]

Supplementary Video 1. In a single limb, a vessel looped back on itself and pointed down the leg before appearing to merge with another vessel travelling up the leg (also shown in Figure 3). This was evidently the direction of flow for this vessel, and not reflux, as multiple pumping events were clearly observed in the vessel over the course of four minutes (30 second video shown for brevity). Interestingly, this individual also demonstrated a small tortuous lymphatic vessel segment, which is shown in Figure 4. Note that the orientation of this video matches Figure 3. in the main manuscript, with the superior aspect of the limb at the top of the frame and the inferior at the bottom. An exposure time of 40ms was employed when recording this video.

Supplementary Video 2. A 34 second clip of a 42-year-old female volunteer’s leg in which lymph containing Indocyanine Green could be forced back down the limb (toward the top of the frame) by applying pressure to the limb. This suggests incompetent valves within this individual’s lower limb lymphatics. Once the hand is removed, the lymphatic fluid rapidly flows back up the limb. A similar finding was noted for both lower limbs. A high exposure time (167ms) was required to visualise the lymphatics in this video reducing the temporal resolution to ~6 frames per second.
